# Analysis of Surface Microgeometry Created by Electric Discharge Machining

**DOI:** 10.3390/ma13173830

**Published:** 2020-08-30

**Authors:** Tomasz Bartkowiak, Michał Mendak, Krzysztof Mrozek, Michał Wieczorowski

**Affiliations:** Institute of Mechanical Technology, Poznan University of Technology, 60-965 Poznań, Poland; michal.mendak@put.poznan.pl (M.M.); krzysztof.mrozek@put.poznan.pl (K.M.); michal.wieczorowski@put.poznan.pl (M.W.)

**Keywords:** EDM, craters, multiscale analysis, surface topography, microgeometry

## Abstract

The objective of this work is to study the geometric properties of surface topographies of hot-work tool steel created by electric discharge machining (EDM) using motif and multiscale analysis. The richness of these analyses is tested through calculating the strengths of the correlations between discharge energies and resulting surface characterization parameters, focusing on the most representative surface features—craters, and how they change with scale. Surfaces were created by EDM using estimated energies from 150 to 9468 µJ and measured by focus variation microscope. The measured topographies consist of overlapping microcraters, of which the geometry was characterized using three different analysis: conventional with ISO parameters, and motif and multiscale curvature tensor analysis. Motif analysis uses watershed segmentation which allows extraction and geometrically characterization of each crater. Curvature tensor analysis focuses on the characterization of principal curvatures and their function and their evolution with scale. Strong correlations (*R*^2^ > 0.9) were observed between craters height, diameter, area and curvature using linear and logarithmic regressions. Conventional areal parameter related to heights dispersion were found to correlate stronger using logarithmic regression. Geometric characterization of process-specific topographic formations is considered to be a natural and intuitive way of analyzing the complexity of studied surfaces. The presented approach allows extraction of information directly relating to the shape and size of topographic features of interest. In the tested conditions, the surface finish is mostly affected and potentially controlled by discharge energy at larger scales which is associated with sizes of fabricated craters.

## 1. Introduction

The topography of a manufactured surface is a direct product of the physical phenomena occurring during its formation, and contains information critical to comprehend and reconstruct what happened. The investigation of the representative surface features created by the manufacturing process is, therefore, particularly valuable for those processes which are still not fully understood. This involves EDM (electric discharge machining), in which energy is transferred between tool and workpiece via electric discharge.

There exist multiple ways of describing surface topography of engineered surfaces. The most typical approach is the characterization by ISO 25178-2 areal texture parameters [1] calculated for the measured regions after form removal and filtration. Those analyses are aimed at quantifying the properties of an entire analyzed region as a set of texture parameters, which are mostly based on statistical measures such as average, standard deviation or rms (root mean square) [2]. Another approach is to describe process-specific topographic features which are inherent to their fabrication such as ridges and valleys for milling or turning, and craters for EDM. Some feature-based parameters, included in ISO standard, are potentially relevant for characterization of the formations created by electric discharge machining. However, they do not provide the information about the scales of those features. In other words, topographic features of a given size are best discernible when observed at particular scales. This phenomenon is the principle of the third approach, i.e., multiscale methods [3]. The importance of scale lies in the characterizations of physical interactions between formation process and resulted surface topography. These can occur at multiple scales during fabrication.

Geometric quantities of engineered surfaces, such as length [4] or area [5] usually change with the scale of observation. Recently developed multiscale curvature method allows studying the evolution of principle, mean and Gaussian curvatures calculated at a given location on the surface with scale. These measures are indicative of local shape, e.g., can determine concavity or convexity and the amount by which surface bends in particular directions [6,7].

In EDM, fabricated surface can be conceived as a composition of craters and plateaus, which individual geometric properties, i.e., depth, radius, volume and curvature strongly depend on processing parameters. Knowledge about the correlations between formation and surface topographies, i.e., roughness or finish, is vital for process design and control [8,9]. There are essentially two components of the value added to a workpiece by the EDM process: form and surface finish. Those can be controlled by technological parameters (e.g., voltage, current, pulse time and many more), electrode material and shape and dielectric fluid [10,11]. There is probable not a single universal technological parameter which can be used to determine the microgeometry of resulted surface. Craters dimensions strongly depend on the amount of energy that is transferred from the electrode to the workpiece via electric discharges, in a stochastic manner. Klocke et al. found that the depth of recast layer was influenced by resistance and capacity in circuit, both of which impact on the discharge energy and higher energy led to thicker recast layer [12]. Giridharan et al. developed so-called “anode model” in which the energy that reaches the workpiece and forms the crater is proportional to the discharge energy [13]. The presented numerical and experimental results were shown to be in a good agreement in terms of crater morphology. Other studies have indicated proportional relations between discharge and crater volume [14], area [15], diameter [16] or size [17]. Ding et al. showed that in micro wire electrical discharge machining, the spark energy directly influenced both the average diameter and the maximum depth of craters. They found that relationship to follow a logarithmic trend [18]. Some studies indicate linear relations between craters diameter and depth [14,19]. All of those studies show strong functional relations between certain crater dimensions and discharge energy for constant material properties (e.g., physical properties of electrode, workpiece and dielectric fluid). This study concentrates on the energy of electric discharge as a unifying technological parameter that strongly correlates with microgeometry of resulted surface topographies taking into account the above assumption.

In the literature, surface topographies are often characterized using basic ISO parameters: average roughness, *R*a (profile) or *S*a (areal) [1]. Those parameters were mostly developed for an analysis of conventionally machined surfaces (milled, turned or ground) and they lack the ability to exploit the complexity of non-traditionally manufactured surfaces [2]. They were used in EDM to show the affect of smaller pulse duration on the creation of smaller craters, characterized by lower values of *R*a [11]. Masuzawa et al. showed that low open circuit voltage produced small craters and, hence, lesser surface roughness expressed also in average roughness [20]. Guu stated that greater pulse currents and longer pulse durations produced textures of higher *R*a. [21]. In micro-EDM, average areal roughness (*S*a) seemed to correlate strongly with discharge energy [15]. Bäckemo et al. created a predictive computer supported model of surface topography created as a result of impacts caused by electric discharges between metallic substrate and an electrode [22]. They found that the roughness parameters followed an inverse exponential trend as a function of impact number, and that the strongly concave curvatures reached equilibrium at an earlier impact number for lower depth to radius ratios. The size of each individual impact can depend on the charge that builds up before each spark, which, in turn, is seen as a function of the electric parameters of the process [23]. Klink et al. showed that basic profile roughness parameters are not sufficient to describe the complexity of EDM-created topographies and they proposed the use of average groove width RSm and the average profile gradient *R*Δ*q* for a more sophisticated surface topography description [24].

The traditional height parameters do not consider the spacing nor the sequence of the heights, as well as they do not characterize characteristic surface features. The development of feature parameters addresses the latter. These parameters rely on a technique called *segmentation*, which is based upon the application of a watershed algorithm [25], associated with an algorithm for simplifying graphs that describe the relationships between individual points [26]. Segmentation is useful in identifying significant peaks and pits, and can be used for calculating peak density and peak curvature. In addition, specific parameters were created to quantify the area and mean volume of motifs identified by segmentation, distinguishing between open and closed motifs, depending upon whether or not they are in contact with the edge of the microscopic image. Such distinction is necessary, since open motifs do not provide full information about a particular crater that they describe. This analysis appears to be a prospective candidate to analyze geometric quantities of craters.

Electric discharge machining is a manufacturing process in which material is removed from the workpiece by a series of rapidly recurring current discharges between a tool and workpiece electrodes, separated by a dielectric fluid (liquid or gas) and in response to voltage pulses. The physical phenomenon occurring between the electrodes in EDM when manufacturing surface features on the micrometer scale is not entirely understood [27]. For short pulses and energies, there is not enough time for material to be adequately heated for removal and therefore almost none takes place. In that case, electrostatic force which acts on the surface becomes an essential factor in the removal of metal for short pulses [28]. The acquisitions of the resulting surface topographies and the multiscale analysis of the geometry of created microfeatures by EDM can provide evidence of the material response to the discharge [15]. Relations between curvature and discharge energies (between 18 and 16,500 nJ) in micro-EDM were studied by Bartkowiak and Brown [7]. They also suggested, by having analyzed the principal, mean and Gaussian curvature, that different formation processes governed the creation of surfaces created by higher energies.

This study aims at characterizing the geometric properties of fabricated surface topographies in micrometer scales. This is demonstrated by the use of motif and multiscale analysis to characterize surfaces of hot-work tool steel created by electric discharge machining (EDM), and then to study correlations between the discharge energies and the resulting surface topographies, focusing on microgeometry of craters. In particular, the strengths of the correlations (*R*^2^) between motif and curvature characterizations (i.e., principal, Gaussian or mean curvature) and discharge energies is sought as a function of scale. Motifs are used here to derive geometrical properties of created craters (e.g., area, depth and diameter), whereas curvature allows characterization of their shapes in multiple scales. The proposed approach is feature-based and focuses on the geometric specificity of the topographies created by EDM. As a comparison, additional conventional analysis of surface texture using ISO 25178 standard and its areal characterization parameters are performed. Geometric characterizations of process-specific topographic objects is considered to be a natural and intuitive way of analysis the complexity of EDM surfaces. In contrast to analysis of surface topography through areal texture parameters (as in ISO 25178 2), the presented approach allows extraction of information directly relating to the shape and size of topographic features of interest. The richness of this information is tested via correlations with processing parameter.

The approach here is to calculate the curvature tensors on a surface as functions of position and scale. Statistical characterization parameters of principal, mean and Gaussian are compared with the pulse energies in EDM as calculated from technological parameters. This is accompanied with motif analyses, in which the height or depth, diameter, area and volume of detected motifs are evaluated. According to our best knowledge, this is the first study, in which areal motifs are used to characterize the topographic features created by EDM. Other studies that covered some geometrical aspects of craters, used manual measurements of those quantities from measured datasets. Motif and curvature analysis is supplemented with a comprehensive study using ISO 25178 standard areal parameters, in the analysis of process–surface-texture interactions in this manufacturing process. The differences and similarities between those methods are discussed with a reference to discharge energies and characterization capabilities. Application of both methods reveals relations between microgeometry of surface topographies and formation process, which is impossible to be observed using a conventional approach.

## 2. Materials and Methods

### 2.1. Samples Preparation

**Figure 1:**
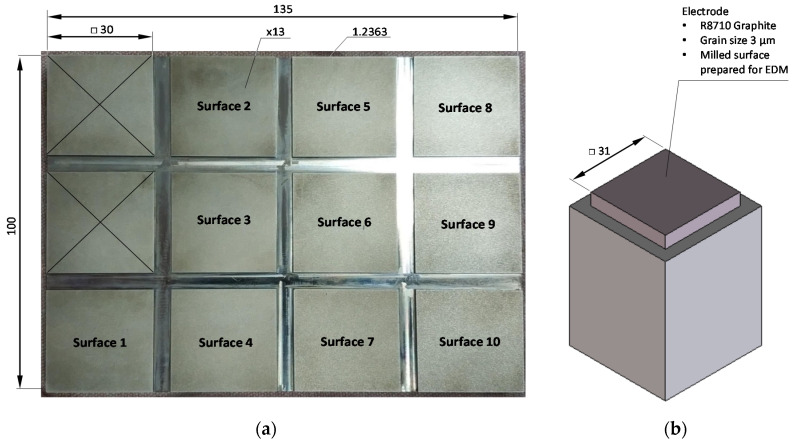
Materials prepared for the study: (**a**) 52 HRC workpiece made of 1.2363 steel as machined; (**b**) graphite electrode with 3 µm grain size.

In this work, roughing and finishing EDM processes of twelve flat surfaces were performed (Figure 1a) with different technological parameters. Machined surfaces were prepared separately with dimensions of 30 mm × 30 mm and height of 3 mm on a 1.2363/X100CrMoV5 (EN 10027-2/ISO4957) on a steel block hardened to 52 HRC, with dimensions of 135 mm × 100 mm × 13 mm. Chemical composition of the steel used in the study is shown in Table 1. Hardness was tested prior to machining, using Rockwell method using C scale and N3A testing machine (EMCO-TEST Prüfmaschinen GmbH, Kuchl, Austria) equipped with 120° diamond spheroconical intender. Minor load was set to 98.07 N and load was 1471.0 N.

Prior to EDM, the twelve surfaces and base area of the steel block were ground to a height of 13 ± 0.01 mm. The purpose of this process is to obtain the same initial surface texture and height of the workpiece for each of the machined surfaces. Basic areal height parameters according to ISO 25178 2 of as-ground surfaces are shown in Table 2. Waviness and roughness parameters were calculated using robust Gaussian filter with cutoff wavelength of 0.25 mm applied to each of primary surfaces after levelling. Five measurements were done at representative regions using a focus variation microscope (FVM)—Alicona Infinite Focus G5 (Alicona Imaging GmbH, Raaba/Graz, Austria).

Each surface was then machined with two types of electrodes—roughing and finishing, which had identical geometric parameters (Figure 1b). A total of 24 graphite electrodes with the same geometric and material characteristics were made for the experiment (see physical properties in Table A1 (Appendix A). Two of the dozen created surfaces were made with test parameters and were excluded from the further analysis.

**Figure 2:**
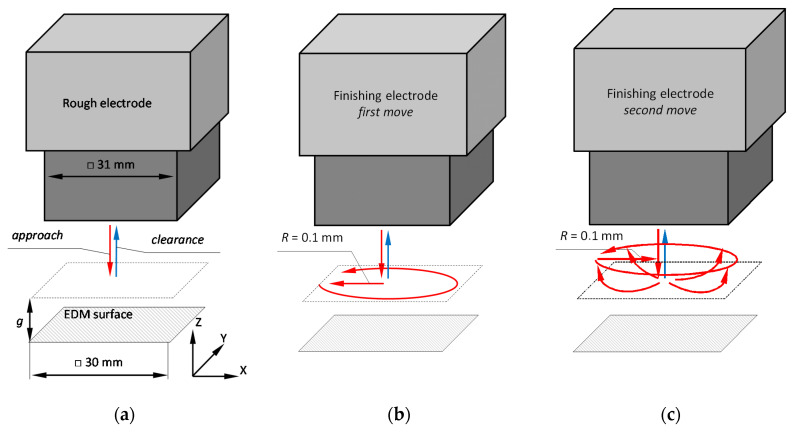
Experiment: (**a**) rough EDM; (**b**) finishing EDM—first move; (**c**) finishing EDM—second move.

The electric discharge machining was conducted using the high-end EDM die-sinking machine Form X400 (Agie Charmilles, Losone, Switzerland) and was divided into two stages (see Table A2 in Appendix A for machine parameters and dielectric fluid). In the first roughing stage, 1.5 mm layer of material was removed in the *z*-direction (Figure 2a). This reflects to the standard industrial process in which allowances for this material and geometry are chosen by machine tool control unit automatically to ensure that any residual form, waviness and roughness is fully removed. For as-ground surfaces maximum height (*S*z) for waviness and roughness is two orders of magnitude lower than layers removed by electric discharge machining. During the rough EDM process the electrode movement was limited to the *z*-axis as reciprocating motions. There were two types of movement in *z*-direction—the working travel, which allows the tool electrode to get closer to the machined material and to let the electrical discharges occur, and the idle travel, during which flushing and removal of the eroded material (by-products) from the spark gap take place. The second stage of the processing was finishing, which consisted of two types of movements (Figure 2b,c). The first one was a motion on the *xy* plane in a circle with a diameter of 0.1 mm above the machined surface. After removal of subsequent layers of material, the tool electrode moved forward along *z*-axis and repeated circular motion. The second type was a motion in a circular trajectory with a diameter of 0.1 mm on planes perpendicular to the *xy* plane. In both EDM processes, a commercially available synthetic hydrocarbon fluid (108 MP-SE by Novotec BV, Reuver, the Netherlands) was used.

In this study, a primary focus is given on the surface topography in relation to the technological parameters of the finishing process. The most important parameters of the process for each of ten analyzed surfaces (S1–S10) are included in Table 3. These include: spark voltage (*U*), current (*I*), on- and off-time (*T*_on_ and *T*_off_) as well as face and side gap. Based on the given technological parameters we estimate the discharge energy by applying the formula:

(1) $$E=U\cdot I\cdot T_{on},$$

Without considering the actual values of voltage and current over time. This was done for the practical reason as a commercial EDM machine tool either does not measure those quantities over time or simply does not allow to extract this kind of information directly from the control unit without interfering with hardware and software. Still those technological parameters can be adjusted to achieve different surface finish. The approach to calculate the energy straight from technological parameters of pulses as offered by the control system of EDM machine was also done by Ramasawmy and Blunt [29]. Although, no information about the exact values of voltage and current over time is given, it seems not to interfere with achieving strong correlations with areal texture parameters. This might suggest that this effect is either compensated internally by control system, e.g., by averaging at least one technological parameter, or that it is similar for all discharge energies.

In this process, they were selected to achieve different surface finish according to VDI 3400 standard. This standard distinguishes 45 classes (VDI K) depending on average roughness (*R*a) which can be determined by the following formula:

(2) VDI K=20·lg(10·Ra).

In the presented case, processing parameters were determined automatically by the machine tool control system to obtain surface finish between VDI 16 and VDI 25 for a given electrode (size and material) and workpiece (material). Those classes were chosen to maintain the constant polarity during machining and to achieve different topographies. An operator cannot manipulate any detailed parameters of electric discharge for a selected surface finish. This study reflects the real industrial process and the presented tests were performed in a manner corresponding to the technological processes as indicated by the machine tool manufacturer.

Due to the financial constraints of this study only a single sample was manufactured. However, the stability of the discussed EDM process can be supported by the fact that the machine tool produces inserts for injection molding on a regular basis. Those are regularly tested by the owner of the machine so to check their dimensional and surface quality. The machine is fairly new, and is high-end and well-maintained, which can also testify for the very probable replicability of the results.

### 2.2. Measurements of Surface Topography

Surfaces (S1 to S10) were measured with a focus variation microscope (FVM), Alicona Infinite Focus G5, using Single Imagefield mode and a 50× objective. Measurement settings are presented in Table 4. In order to handle a potential non-homogeneity of the results, each surface was measured at five independent locations in a cross-like pattern with the central area located in the center of the machined surface. A combination of coaxial light source and an external lighting was used, making only slight adjustments, as lighting conditions varied among the measured surfaces. Surfaces were treated with isopropyl alcohol to remove dielectric fluid and dust.

Each measured surface underwent the following steps, using the exact same processing parameters, therefore producing comparable results:

- Dataset leveling using least squares method;
- Outliers removal;
- Filling in the non-measured points [30];
- Calculation of areal ISO standard parameters, curvature tensor analysis and motif analysis from primary surface;
- Extraction of roughness and waviness surface with gaussian filter;
- Motif analysis and areal parameters extraction from both roughness and waviness surface.

**Figure 3:**
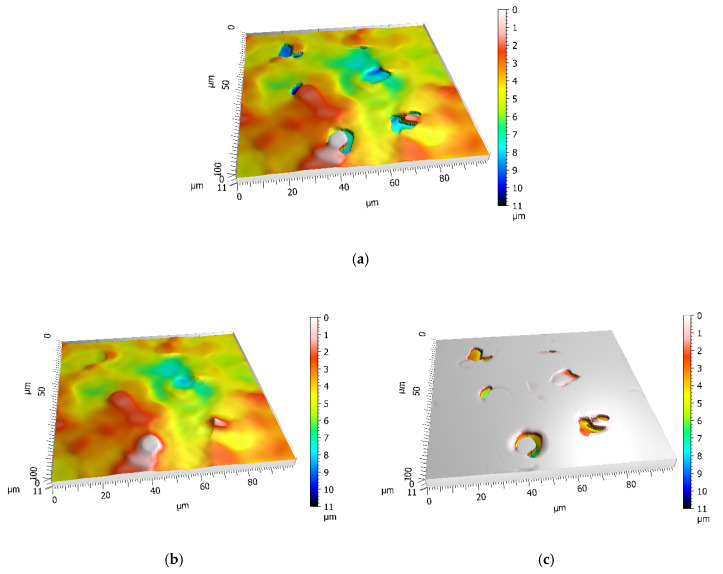
Morphological surface filtration: (**a**) raw surface; (**b**) after filtration using 16μm sphere, closing filter; (**c**) residue surface, all outliers clearly visible.

In FVM, height registration is based on contrast estimation from a given region, providing a proper lighting is used [31]. When measuring surfaces with smooth, reflective areas the FVM often miscalculates the height of the surface resulting in “the plateau-like formation”, as described by Senin et al. [32]. The same effect was observed in this study and the example is presented on Figure 3a. Therefore, the raw measurement (Figure 3a), had to be subjected to preliminary filtration in order to remove the plateau-like artefacts. Three different approaches were considered: threshold method, robust gaussian filter and morphological filter. The choice of these three methods was dictated by both budget and software limitations. Nevertheless, various filtration methods available in the MountainsMap software were tested, including spatial and standard filters. The three aforementioned methods yielded most promising results, with the least significant, or even negligible, effect on standard roughness parameters. The first method, which was based on a manual choice of lower and upper thresholds for each surface, did remove the plateau-like regions, leaving just the steep slopes, which could be later removed using a dedicated outlier removal technique. Areas that were filtered out were replaced with non-measured points, which were then filled as a smooth shape (calculated from neighboring points). This method was discarded as, for several datasets, a significant portion of other pits from primary surface was also removed, resulting in disqualifying distortions at the later stage of the analysis. The other method utilized the robust gaussian filtration, which is characterized by its mean plane following the general trend of the surface, without being affected by outliers. Its use in extraction of surface features was extensively examined by Lou et al. [33]. In that study it proved to be ineligible, because it either overly smoothed the surface out or failed to exclude the outliers. The last method was a morphological closing filter using sphere with 16 μm diameter. This value was set intentionally as it compromised between artefacts filtration and good surface mapping. The effects of using such filter on surface profile is presented in Figure 3b.

The downside of morphological closing filter is, that, on several surfaces, it left a few isolated peaks which had to be removed manually. This could be avoided, by adding the opening operation, however the authors’ intention was to limit the influence of outliers filtration on the measured surface. No additional form or noise removal was used. Form removal was not applicable due to relatively small measurement area and it was feared it might influence the craters geometry representation. Only levelling using least squares method was applied.

There is little consistency regarding surface or profile filtration among researchers, partly due to lack of information on the filters, cut-off lengths or nesting index used to extract waviness and roughness profile. Some researchers [34,35] provided the Ls or cut-off length (2.5 mm) and the standards name. Some [24] simply stated compliance with the standard.

Areal parameters do not differentiate between roughness, waviness or even primary surface, and therefore a nesting index value must be stated before presenting the results. In this analysis, all three types surfaces were taken into account. As stated by Townsend et al. [36], “filtering is based on the roughness or scale of the largest significant feature”. In case of the predicted and calculated roughness values, it indicated that the correct nesting index value should be 0.25 mm. Choosing a smaller value would lead to an excess transfer of roughness information into the waviness surface. The filtering is required, as further stated by Townsend et al. [36], because of significant variations between the results of filtered and unfiltered surface. Given the relatively small measurement area, which limits the value of nesting index, an unfiltered surface was also subjected to areal parameter analysis.

### 2.3. Analysis of Microgeometry

In this study, each surface is treated as a composition of craters and plateaus resulted from electric discharging with electrode. In order to fully characterize the geometry of those features, three types of analyses were conducted: conventional approach with ISO standard areal parameters, motif analysis and multiscale curvature tensor analysis. The last two directly focus on the characterization of geometrical features or parameters of surface.

#### 2.3.1. Conventional Approach with ISO Parameters

Influence of EDM parameters on surface topography was studied by numerous authors. Most publications concern profile roughness [24,29,34,37,38], and only few considered areal parameters [29,35] (mainly the height class, e.g., Sa or Sq), analogical to the most widely used profile roughness characteristics. Most of them considered at least three parameters, and two examined just one [34,39]. The complexity of EDM surfaces may require more complex analysis to comprehensively study all aspects of their topography. For this analysis, MontainsMap software (version 7, Digital Surf, Besancon, France) is used to calculate areal parameters described in ISO 25178, divided into seven groups:

- Height, which is a class of parameters, that quantify the information on the *z*-axis of the surface;
- Functional, derived from the Abbott–Firestone curve, which describes the height cumulative distribution on the surface;
- Spatial, which describe topographic characteristics and quantify the lateral information of the surface;
- Hybrid, a class of surface finish parameters, that consider both the amplitude and spacing between heights;
- Functional (volume), which involves volume parameters calculated from the Abbott-Firestone curve;
- Feature, derived from the segmentation of surface into motifs; and
- Functional (stratified surfaces), which includes parameters designed for automotive industry, considering certain aspects of a surface interactions, such as lubrication and grinding.

All parameters from those groups, used in this study are presented in Table 5. In study, a special focus is given to height, feature and functional groups. Height parameters are the most widely used in the academia and the industry, as they are the most intuitive and simple to calculate for given dataset. These parameters, such as Sa (average roughness) correlated strongly with technological parameters in EDM [15]. Feature parameters characterize geometrical properties of motifs which may be associated with craters or dales created by electric discharge. Functional parameters allow to characterize geometric parameter—volume of the void and material with respect to surface core, peaks or valleys, what might be associated with craters [40].

#### 2.3.2. Motif Analysis

Different manufacturing techniques lead to creation of a vast variety of surface textures, each having its own unique artefacts and characteristics. In EDM, one of the characteristic features of the manufactured surface is a crater, created by electric discharge. This kind of features are hard to examine using standard roughness analysis, although several functional correlations were found [29,35].

The term “motifs” can be used to describe either hills or dales. Its extreme points are called peaks and pits, respectively. They are limited by lines called course (hills) or ridge line (dales). Motifs are established using method called segmentation, which utilizes a watershed algorithm [25].

This method is commonly used by researchers, mainly in the analysis of surfaces created by additive manufacturing techniques [32,33,36] and in applications, where grain or fault detection is desired, such as protruding diamond grains in grinding wheels [41].

In this study, watershed segmentation and motifs analysis were used to identify craters and perform a morphological characterization of the detected features, including:

- Height—distance between the lowest saddle point and pit;
- Area—horizontal area limited by the ridge line;
- Volume—volume of the void below the plane of the lowest saddle point;
- Equivalent diameter—diameter of a disk which area is equal to that of a grain;
- Mean diameter—average diameter of a disc constructed at the center of the gravity of a grain.

There was no pre-processing (areal filter) used. Pruning criteria were established as follows:

- Height—<0.75% Sz (maximum height),
- Area—<0.25% of surface area

The above values provided the most consistent and reliable segmentation, without signs of “over-“ or “under-segmentation”, where visual boundaries of the craters were consistent with calculated ridge lines. Calculation method was set to pit detection using MountainsMap 7 software.

#### 2.3.3. Multiscale Curvature Tensor Analysis

In general, the curvature tensor *T* is a symmetric 3 × 3 matrix that can be expressed as a product:

(3) T=D·P·D,

where:$\;P=\left(k_{1},k_{1},n\right)$ and

(4) $$D=\left[\begin{array}{ccc} \kappa_{1}\; & 0 & 0\\ 0 & \kappa_{2} & 0\\ 0 & 0 & 0 \end{array}\right],$$

The eigenvalues *κ*_1_ and *κ*_2_ represent the principal curvatures, maximal and minimal magnitudes respectively. The sign is used to designate concave surfaces as positive and convex as negative. The eigenvectors ***k*_1_**, ***k*_2_** are the corresponding principal directions of maximum and minimum curvature and *n* is the surface normal unit vector at the location of the calculated curvature. Mean (*H*) and Gaussian curvature (*K*) can be calculated from principle curvatures.

Measurement data is usually a discrete set of point coordinates described in Cartesian coordinate system. In this paper, the curvature tensor is estimated from datasets with regular spacing in x- and y-direction.

The entire concept of multiscale curvature method bases on the principle that the shape of objects, as well as their other geometric characteristics, depend on the scale of observation. For finer scales, surfaces seem to contain more geometrically complex details which curvature is high, whereas for larger scales, they appear flat or only form is visible. Considering the analyzed datasets, the geometry of craters should manifest itself at particular ranges of scales associated with their sizes. This is examined by calculation of curvature tensor at each location for range of scales between 0.352 and 13.716 µm.

The result of multiscale curvature tensor analysis is a curvature tensor calculated for each triangular patch whose size is scale-dependent. As presented in [42], distributions of maximum, minimum, mean and Gaussian curvature can be derived at a particular scale and simple statistical measures can be calculated from that distribution. These include average (signed with “a”) and standard deviation (with “q”) of curvature distributions: κ1a, κ1q, κ2a, κ2q, Ha, Hq, Ka and Kq. They describe central tendencies and variabilities including sign of curvature (positive or negative). In this work, new characterization parameters are proposed which quantify curvature distribution regardless the sign by taking absolute values. They are defined at a particular scale *s* as below:

- $\kappa_{1}a_{\mathrm{abs}}$—average absolute maximum curvature
  (5) $$\kappa_{1}a_{\mathrm{abs}}=\frac{1}{n}\overset{n}{\underset{i=1}{\sum }}\left|\kappa_{1i}\right|,$$
- $\kappa_{1}q_{\mathrm{abs}}$—standard deviation of absolute maximum curvature
  (6) $$\kappa_{1}q_{\mathrm{abs}}=\sqrt{\frac{\sum_{i=1}^{n}{\left(\left|\kappa_{1i}\right|-\kappa_{1}a_{\mathrm{abs}}\right)}^{2}}{n}},$$
- $\kappa_{2}a_{\mathrm{abs}}$—average absolute minimum curvature
  (7) $$\kappa_{2}a_{\mathrm{abs}}=\frac{1}{n}\overset{n}{\underset{i=1}{\sum }}\left|\kappa_{2i}\right|,$$
- $\kappa_{2}q_{\mathrm{abs}}$—standard deviation of absolute minimum curvature
  (8) $$\kappa_{2}q_{\mathrm{abs}}=\sqrt{\frac{\sum_{i=1}^{n}{\left(\left|\kappa_{2i}\right|-\kappa_{2}a_{abs}\right)}^{2}}{n}},$$
- ${\mathrm{Ha}}_{\mathrm{abs}}$—average absolute mean curvature
  (9) $${\mathrm{Ha}}_{\mathrm{abs}}=\frac{1}{n}\overset{n}{\underset{i=1}{\sum }}\left|H_{i}\right|$$
- ${\mathrm{Hq}}_{\mathrm{abs}}$—standard deviation of absolute mean curvature
  (10) $${\mathrm{Hq}}_{\mathrm{abs}}=\sqrt{\frac{\sum_{i=1}^{n}{\left(\left|H_{i}\right|-{\mathrm{Ha}}_{abs}\right)}^{2}}{n},}$$
- ${\mathrm{Ka}}_{\mathrm{abs}}\;$—average absolute Gaussian curvature
  (11) $${\mathrm{Ka}}_{\mathrm{abs}}=\frac{1}{n}\overset{n}{\underset{i=1}{\sum }}\left|K_{i}\right|,$$
- ${\mathrm{Kq}}_{\mathrm{abs}}$—standard deviation of absolute Gaussian curvature
  (12) $${\mathrm{Kq}}_{\mathrm{abs}}=\sqrt{\frac{\sum_{i=1}^{n}{\left(\left|K_{i}\right|-{\mathrm{Ka}}_{\mathrm{abs}}\right)}^{2}}{n}}.$$
  where *i* symbol corresponds to a particular *i*-patch for which the curvature tensor is calculated at scale *s* and *n* is a total number of patches. The scale is associated with the side of the triangular patch for which the curvature is estimated. For the finest scale, it is equal to the original sampling interval in *x* or *y* (they are both equal in the presented measurements). With the increasing scale, the dimensions of the triangular patch increase as well. The detailed procedure of how to calculate curvature tensor in multiple scales is shown in [6,7,42].

In this work, parameters (3)–(10) as well as other given in [42], were calculated for a series of scales and regressed linearly and logarithmically with discharge energy. All curvature computations were performed using Wolfram Mathematica software (version 12, Wolfram Research, Oxfordshire, UK).

## 3. Results

This part is structured in four subsections. The first subsection is dedicated to the measurements of surface textures and their visual impressions of topographic structures as a function of discharge energy. This is followed by the subsequent subsection focusing on ISO standard areal parameters. Not all the parameters are entirely related to the geometric characterization of manufactured features. However, they are most widely used and well understood by the academia and industry, therefore they deserve their place in the characterization of EDMed surfaces in relation to their fabrication parameters. Yet their ability to analyze the microgeometry of created surface features, i.e., craters, is limited. Thus, results of two novel methods are introduced in the next two subsections: motifs and multiscale curvature analysis. They focus on the geometric quantities (area, diameter, height, volume, curvature) and are believed to better describe the nature of interactions between physical phenomena occurring during machining and resulted topographies. Every presented characterization parameter is analyzed with respect to the discharge energy.

### 3.1. Measurements

**Figure 4:**
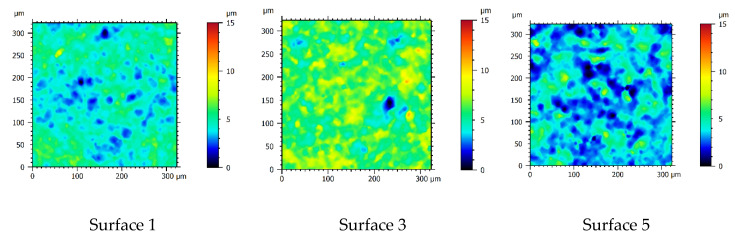

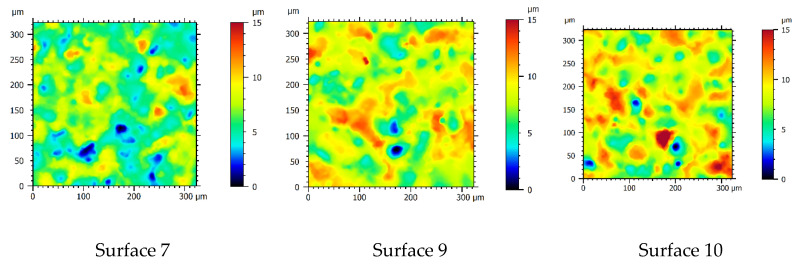
Six out of ten representatives of measured surfaces randomly chosen after dataset preparation step. Renderings of other surfaces are available in Supplementary Materials (Figure S1).

Renderings of the measured regions (six exemplary regions from S1, S3, S5, S7, S9 and S10) created using different technological parameters are depicted in Figure 4. The peak to valley roughness clearly increases with the discharge energy.

A total of 50 measurements were performed on 10 test surfaces using a focus variation microscope with setup parameters described in Section 2.2. Figure 4 depicts each measured surface after dataset preparation process. For comparison measures all color scales on vertical axes were scaled up to the same range. There are evident discrepancies between the surface topographies, which might be associated with different discharge energies. Craters become larger (in x- and y-directions) and there are more significant differences between minimal and maximal height value (z-direction).

### 3.2. ISO Parameters

**Figure 5:**
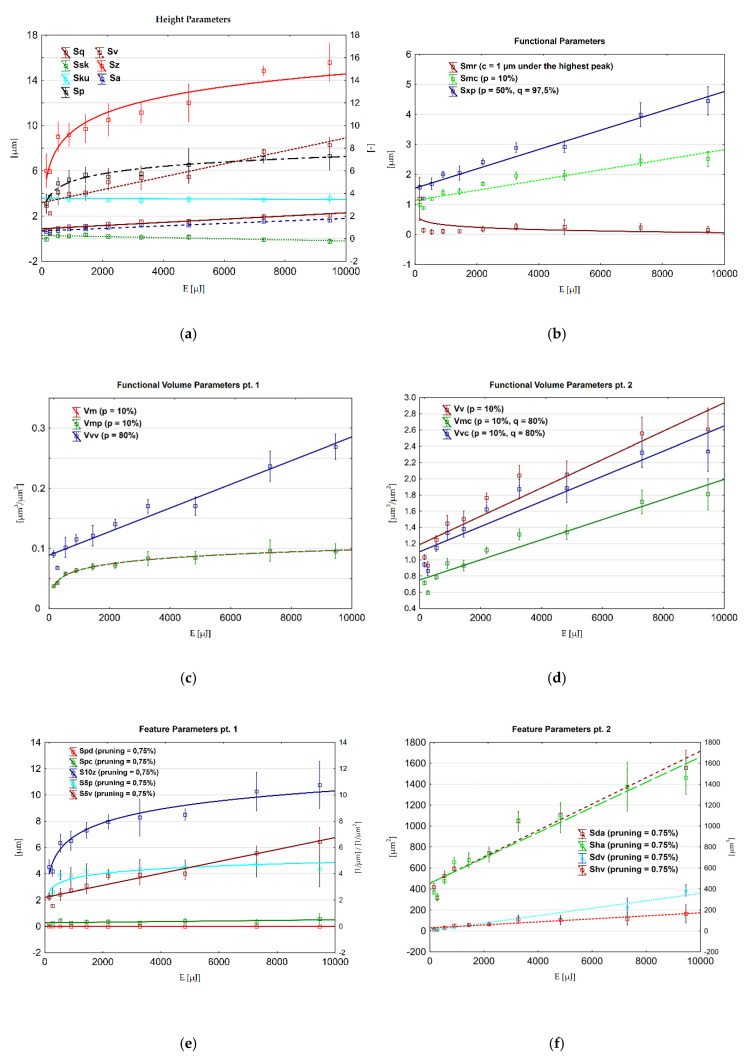
Scatter plots of ISO 25178 areal parameters correlated with discharge energies using linear or logarithmic regression. (**a**) height ; (**b**) functional ; (**c**,**d**) functional volume; (**e**) and (**f**) feature parameters.

As described in Section 2.3.1, a primary focus is given on three groups of areal parameters. These were presented in were calculated from five measured regions per each surface. Figure 5 presents the scatter plots of selected ISO 25178 areal parameters versus discharge energy value—mean of five measured regions and ±1 standard deviation (SD). Values of coefficient of determination for linear and logarithmic regressions are shown in Table A3 (Appendix B). Presented values are derived from unfiltered surface. The trend lines are also shown to indicate the best fitted functional relations between linear and logarithmic. All ISO 25178 [1] parameters calculated for all ten surfaces are shown in the supplementary spreadsheet to this study.

#### 3.2.1. Height ISO Parameters

Height parameters are in general correlation with discharge energy changes, except for Skewness (Ssk) and Kurtosis (Sku) (Figure 5a). Both of these parameters remained at relatively stable range. Ssk value varied from −0.233 to 0.320 indicating a nearly symmetrical height distribution in relation to the mean plane. Sku varies from 3.005 to 3.867. A higher value (>3) indicates an increased spikiness of the surfaced, which is clearly seen in Figure 5a for S3 (Sku = 3.86).

Basic parameters, such as Sq and Sa changed their values, decreasing from 0.87 or 0.70 μm, respectively, in S1 to 0.799 or 0.63 μm, for S4, and then increasing to 2.23 or 1.75 μm for S10. Sv, apart from a general tendency to increase its value, does not grow with each increment of discharge energy. Both Sz and Sp increase in what appears to be a logarithmic manner. Strong correlations (*R*^2^ > 0.9) were observed for all parameters except for Ssk and Sku. Skewness exhibit the moderate correlation when applying linear regression for an unfiltered surface. No correlations can be noted in residual roughness and waviness surfaces. Sa, Sq and Sv show strongest correlation when calculated using linear regression. Sp and Sz behave similarly for logarithmic regression. Surface S2 seems to deviate significantly from the trend for Sv, but this effect appears to be incidental. Similar observations were made for most of the others, but not all (Vmp, Spc and Spd), ISO and motif parameters. From visual impressions of captured topographies, it seems not to be radically different than S1 or S3. No changes in measurement conditions were also noted for measurements of S2. In order to fully investigate the reason for which S2 deviates significantly from the trend, other samples machined with same parameters would have to be manufactured and analyzed.

Calculated areal average roughness corresponds to the desired VDI surface finish in 5 out of 10 surfaces, with others differing by a single class. It should be emphasized that VDI 3400 standard is widely used in the industry but it was originated in early 1970s, where only available roughness measurement instrument was contact profilometry. Different measurement and filtration techniques can significantly influence the values of surface texture parameters [31,43,44,45]. None of these important issues, for obvious reasons, is addressed in VDI 3400, which may cause direct referencing to surface finish in this standard disputable.

#### 3.2.2. Functional ISO Parameters

Smr depends on the c parameter, while Smc and Sxp depend on p and q. In this study, software default settings for this parameter, i.e., *c* = 1 μm under the highest peak, *p* = 10% for Smc, *p* = 50% and *c* = 97,5% for Sxp were used. For Smr, a steep decline was observed between S1 and S2, followed by flattening of the trend, which may suggest its logarithmic nature. Smc and Sxp also showed a slight decline between S1 and S2. The way these parameters change their values is very similar to that of the functional volume parameters, as seen in Figure 5c,d. Smc and Sxp show strong correlations (*R*^2^ = 0.923 and 0.953, respectively) in both primary (unfiltered) and residual roughness surface.

#### 3.2.3. Spatial ISO Parameters

Selected spatial parameters Sal (autocorrelation length), Str (texture aspect ratio) and Std (texture direction) did not show any significant trends following consecutive increments of discharge energy. This however may be strongly limited by relatively small measurement area. Spatial parameters show poor to medium correlation with discharge energy. The highest values of *R*^2^ were achieved for Str (*R*^2^ = 0.566, linear regression, unfiltered) and Sal (*R*^2^ = 0.815), logarithmic regression, residual roughness).

#### 3.2.4. Hybrid ISO Parameters

Sdq and Sdr show signs of correlation with increased discharge energy. However, for S1 to S3 their values fluctuate and stabilized their inclining trends from S4 to S10. Both parameters correlate strongly with discharge energy in an unfiltered surface, when regressed logarithmically (*R*^2^ = 0.929 and 0.916, respectively). These values slightly decline (*R*^2^ = 0.900 and 0.884), when calculated from residual roughness surface, however both parameters still correlate better for logarithmic regressions.

#### 3.2.5. Functional Volume ISO Parameters

Functional volume parameters depend on the particular value of the p and q parameters. For all, except Vvv, p = 10%, and q = 80% for Vmc and Vvc. The pit void volume (Vvv) was calculated for p = 80%. All functional volume parameters correlated well with discharge energy. A general trend for these parameters is clearly seen on scatter plots in Figure 5c,d. Vm and Vmp also have the highest correlations (*R*^2^ = 0.973 and 0.977, unfiltered and residual roughness, respectively), both regressed logarithmically. All parameters show good correlation (*R*^2^ > 0.9) for both filtered and residual roughness surface.

#### 3.2.6. Feature ISO Parameters

Feature parameters characteristic features which were part of motif analysis (pits, dales, etc.) and therefore pruning settings were set to the same value. The only parameter that did not show any dominating trend is the arithmetic mean peak curvature Spc. Density of peaks increased with the discharged energy in a probable logarithmic manner. S5v and S10z recorded a significant decline in value for S2. However, as the value of S10z increased significantly for the next surface (by almost 50%), the S5v only just regained its value from before the decline. The similar sudden decrease was observed for Sda, Sha and Sdv. The most stable growth was observed for Shv. All parameters, except for Spc, show high values of the coefficient of determination (*R*^2^ > 0.9) for unfiltered surfaces. Some of these values slightly decline, when calculated from residual roughness surface. However, for S5p, Sda and Sha they increased. Shv and S5v declined most significantly after filtration to *R*^2^ = 0.852 and *R*^2^ = 0.888 respectively.

#### 3.2.7. Functional ISO Parameters for Stratified Surfaces

Any significant trend was observed only for three parameters: Sk, Spk, Svk. Core roughness depth Sk showed certain fluctuations of value. There are three clearly visible points of decline (S2, S5 and S8), where the most significant ones are for S2 and S5. The aforementioned parameters exhibit very good correlations in both unfiltered (*R*^2^ > 0.92) and residual roughness surface (*R*^2^ > 0.92)

#### 3.2.8. General Comments on ISO Parameters

Ding et al. modeled wire EDM process using FEM [18]. They noted that craters average diameter and maximum depth as well as height parameter Sa follow logarithmic trend with discharge energy. In this study, as for ISO standard areal parameters, the logarithmic regression shows evidently stronger correlations for areal parameters calculated for unfiltered surfaces (without decomposing to roughness and waviness) which describe peaks distributions (Ssk, Sku, Spd, Smr1), heights (Sp, Sz, Spk) and volumes (Vmp). This comes in the opposition to parameters that describe pits and valleys, for which stronger correlations are found applying linear regression. These differences are not present for both waviness and roughness surfaces filtered using the proposed nesting index, although for some parameters this dependency can be found. Weak relations between Ssk and Sku for differently EDMed surfaces are also noticed by [46,47,48].

### 3.3. Motif Analysis

**Figure 6:**
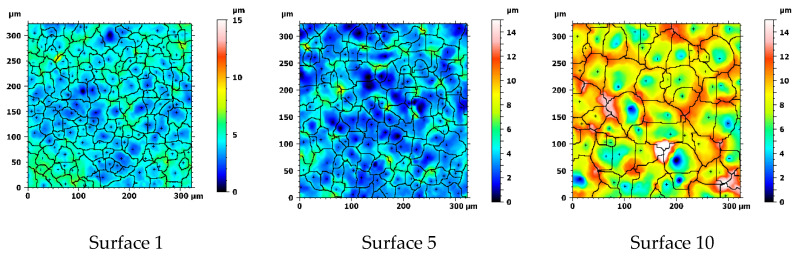
Visualization of effects of the watershed segmentation. Surfaces with watershed boundaries (ridge lines) superimposed on a surface image.

**Figure 7:**
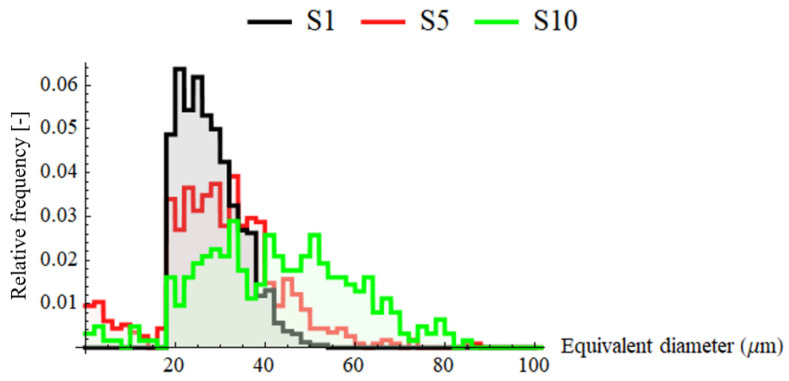
Distributions of equivalent diameters calculated for S1, S5 and S10 surfaces using motif analysis.

Exemplary surfaces S1, S5 and S10, for which watershed segmentation is performed, with ridge lines of the detected motifs can be seen in Figure 6. The pit (extreme point) of each detected dale is marked with a cross. Visually, these segments can be intuitively associated with craters. The distribution of geometrical properties of the segments may reflect the randomness of electric discharges over the machined surface and sudden abruption of material, which lead to creation of overlapping craters which are not perfectly round. This can be visualized using equivalent diameter as presented in Figure 7 in which distributions of that parameter are plotted for S1, S5 and S10. With increasing discharge energy, motifs are on average larger and more dimensionally dispersed. Threshold of 18 µm can be noticed, above which diameters may be associated with craters. This threshold results from the processing parameters during segmentation in which the minimum area of the motif is set. The presence of the smaller motif can occur as the height parameter is a superior criterium or some fine-scale motifs could not be merged.

**Figure 8:**
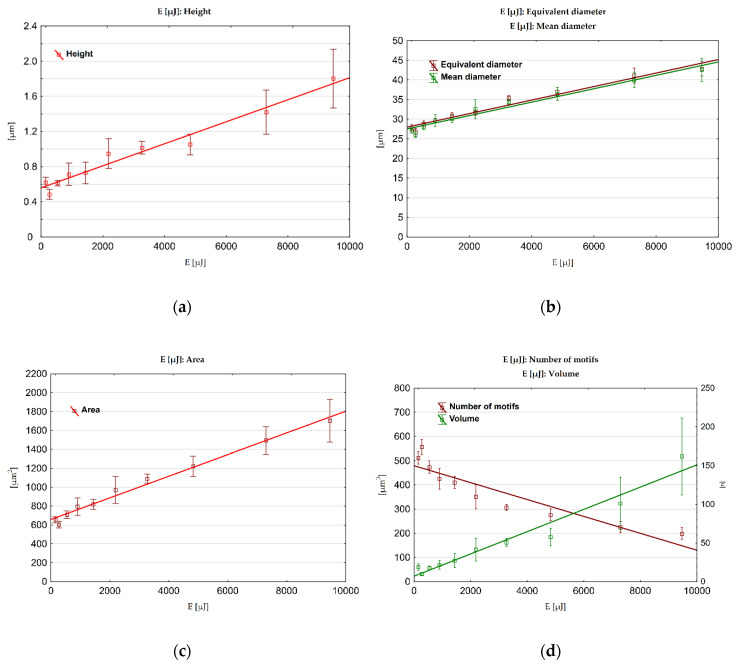
Scatter plots for motif analysis: (**a**) mean height; (**b**) equivalent and mean diameter; (**c**) mean area; (**d**) mean volume and number of motifs correlated with discharge energies using linear regression.

As seen on the scatter plot (Figure 8d), number of motifs decreases as the discharge energy increases. This alone suggests formation of larger, in terms of their volume and diameter, craters on the surface, which conforms to the actual data, presented on other plots on Figure 8b–d. The craters depth or height also increases with the discharge energy. All motifs parameters show a sudden change for S2 what may be considered incidental. All motif parameters, except the number of motifs, exhibit exceptionally strong correlations with discharge energy (*R*^2^ > 0.96) using linear regression. Logarithmic regression seems to be more appropriate to model the functional relation between number of motifs and the energy (*R*^2^ = 0.936). The strongest correlations can be found for mean motif area, for which coefficient of determination is equal to 0.986. Standard deviations of motif geometrical parameters tend to increase with discharge energy. They also show strong correlations when regressed linearly (*R*^2^ > 0.95). This might be interpreted as surfaces machined with higher energies exhibit craters whose dimensions and variability is proportional to this energy. This is also supported by the equivalent diameter distributions presented for two extreme and one middle sets of technological parameters as depicted in Figure 7. More information about the regression analysis can be found in Table A4 (Appendix B).

### 3.4. Multiscale Curvature Analysis

The maximum principal curvature (κ_1_), calculated for two different scales from representative regions located on three different surfaces (S1, S5 and S10) are shown in Figure 10. Negative curvatures (values on the figure) represent convex surface features or peaks. Different scales of calculation show different features. Grooves and pores are visible on κ_1_ curvature plot as positive curvature regions. The amplitude of curvatures decreases with scale what can be explained by the fact that for larger scale features of larger size (and radius) can be characterized. This effect can be observed for other research examples using this method of characterization [6,7,42].

**Figure 9:**
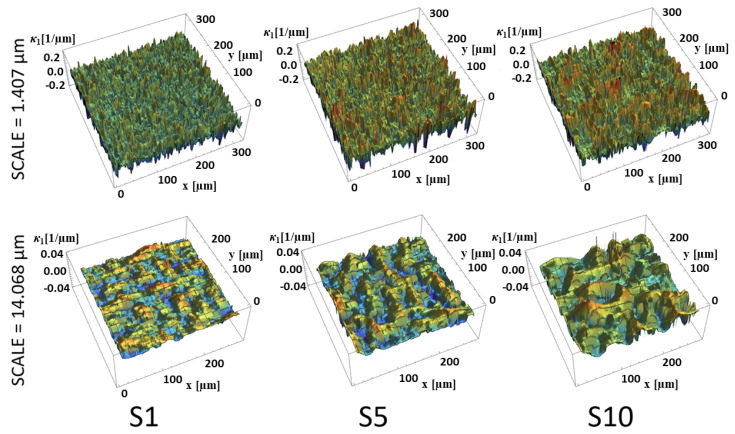
Maximum curvature *κ_1_* for representative measurements of surfaces: S1, S5 and S10, calculated for two different scales: *s* = 1.407 and *s* = 14.068 µm. Please note that vertical scale (*z*-axis) for upper row is fivefold greater than for the lower row.

The calculated values also show the influence of the technological parameters on the surface topography. The maximum curvatures clearly increase with increasing discharge energy for both of the two scales, as shown in Figure 9. However, the differences between curvatures calculated for the smaller scale are less evident in terms of amplitude. The same tendencies were observed for minimum, mean and Gaussian curvatures.

**Figure 10:**
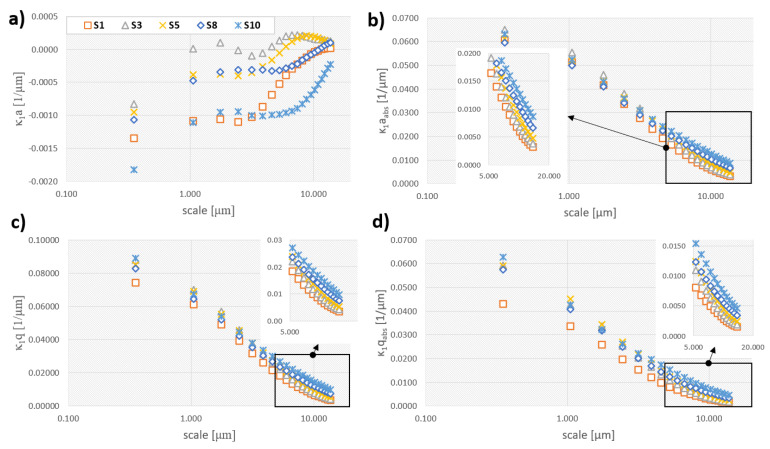
Various statistical parameters related to curvature calculated for surfaces S1, S3, S5, S8 and S10 depicted as a function of scale: (**a**) average of absolute maximum curvature, (**b**) average of absolute maximum curvature, (**c**) standard deviation of maximum curvature, (**d**) standard deviation of absolute maximum curvature.

The mean and standard deviation of the maximum curvature *κ_1_*, as a function of scale, for five representative surfaces S1, S3, S5, S8 and S10 are shown in Figure 10**.** No clear tendency for κ_1_a between surfaces can be observed. These values seem to converge with scale to zero but at different rates, what might be associated with the fact that for the larger scale curvature of form is characterized. Since in all analyzed surface, the form is a flat plane, its curvature is null. This parameter can be associated with average shape (convexity or concavity) at certain scale. Considering averages of absolute values maximum, their values decreases with scale for all surfaces. This parameter characterizes mean deviation from zero (flatness), without taking the signs of curvatures into account. It can be used for describing the evolution of curvature magnitude with scale. Fine-scale features are generally characterized with large curvature and the similar observation is made here as a declining trend. Standard deviation of maximum curvature also decreases with scale. This might be explained by the fact that variation of curvatures declines as the scale gets larger. Similar observation can be made for κ_1_q_abs_. Similar tendencies were noticed for parameters related minimum, mean and Gaussian curvature.

The discharge energy evidently influences the magnitude of the both principal curvatures and their combinations (*H* and *K*). The average parameters (κ_1_a, κ_2_a, Ha and Ka) demonstrated to be least influenced by different material processing as no clear tendency was observed for them for all analyzed scales. Their absolute values and standard deviations are more useful in finding functional relations between process parameter and resulted curvature. This is noticed for scales greater than 5 µm, and might suggest that those topographies can be discriminated at larger scales and that they do not differ significantly at the finest scales.

Two different types of regression (linear and logarithmic) were applied to discharge energy versus three groups of characterization parameters: ISO standard areal parameters, motif analysis parameters and curvature tensor statistical parameters (8). The logarithmic functional relation between discharge and topographic parameters was reported by Ding et al. [18] and it is tested here to confirm or deny it. By strong correlations we take *R*^2^ greater than 0.9.

**Figure 11:**
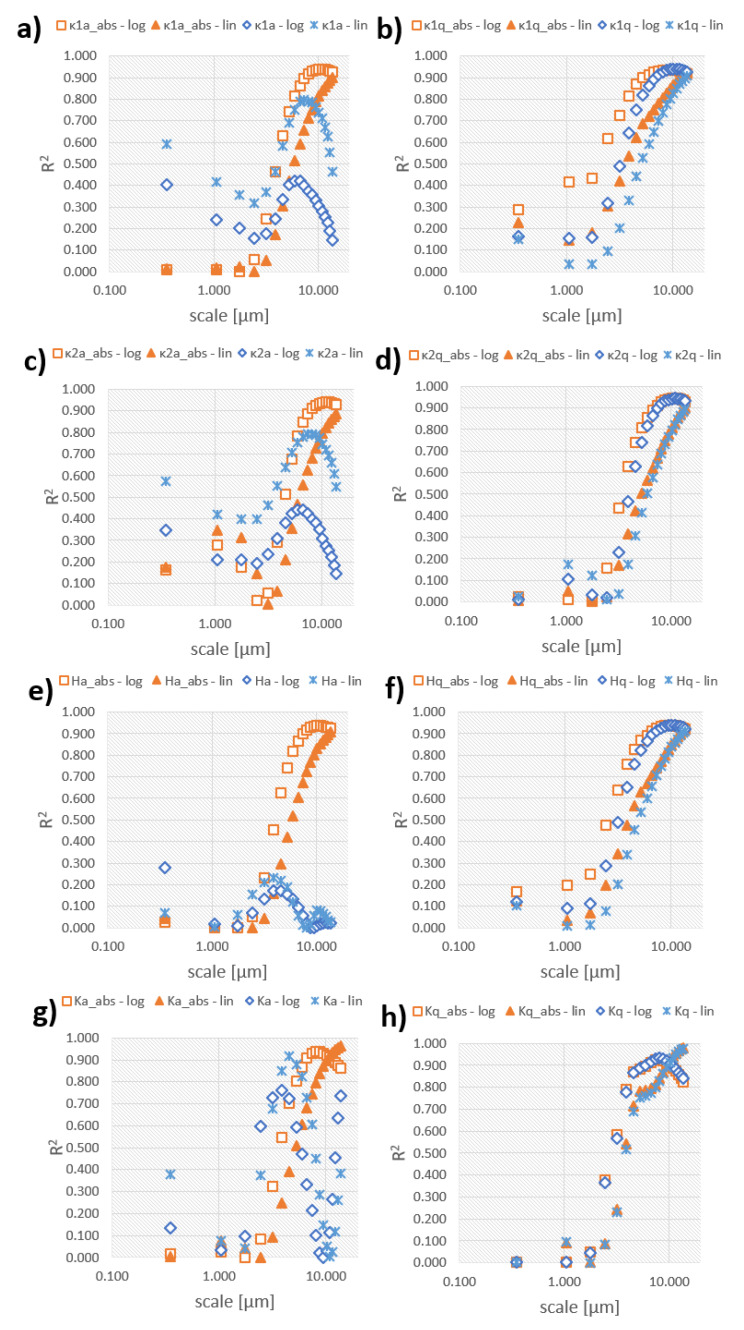
Coefficient of determination for linear and logarithmic regression calculated for statistical parameters calculated for: (**a**) average maximum; (**b**) standard deviation of maximum; (**c**) minimum and (**d**) standard deviation of minimum curvature versus discharge energy; (**e**) average mean curvature; (**f**) standard deviation of mean curvature; (**g**) average Gaussian curvature and (**h**) standard deviation of Gaussian curvature as a function of scale.

The strengths of the linear and logarithmic regression analyses (*R*^2^) for the curvatures versus the discharge energies are shown as a function of scale in Figure 11. Both average principal curvatures κ_1_ and κ_2_ correlate do not correlate well (*R*^2^ < 0.8) with the discharge energy for the analyzed range of scales (Figure 11a). When considering absolute values of curvature, strong correlations are observed for κ_1_a_abs_ and logarithmic regression better than linear reflects that relation as *R*^2^ is greater than 0.9 for *s* > 8.089 µm (total of nine analyzed scales) versus the single largest analyzed scale for linear. Similar tendencies can be noticed for average values of κ_2_ for which only κ_2_a_abs_ correlates strongly when regressed logarithmically for largest scales (>8 µm) (Figure 11c). Standard deviation measures show strong correlations with both models for larger scales (Figure 11b,d). Logarithmic regression correlates for broader range of scales when compared to linear.

The average mean curvature, *H*, correlates well only at larger scales and when absolute values are considered (Figure 11e). Strong correlations are found also for standard deviation but for larger scales as well (Figure 11f). In comparison with other curvature parameters, the Gaussian curvatures, *K*, appear to correlate the best as the largest values of *R*^2^ are found using linear regression for Kq (0.977) and Kq_abs_ (0.981), both at the largest scale equal to 13.716 µm (Figure 11g,h). Ka, as only average non-absolute parameter, corelated strongly but at the middle scale (4.572 µm). Gaussian curvature *K* appeared to correlate the strongest for the widest range of scales. It is the only parameter for which strong correlations were observed when average values are considered. Taking into account the absolute values of curvatures, it significantly improves the strengths of correlations. These parameters might be attributed with the magnitude of curvature, regardless of its sign.

## 4. Discussion

Two types of topographic feature, i.e., craters and ridges, can be distinguished on images rendered from the measurements with the focus variation microscope on the surfaces created by EDM. The features are consistent with known mechanisms in electric discharge machining. The increase of discharge energy lead to the creation of deeper and larger (in area, radius and volume) craters with a greater magnitude of curvature. The measured surfaces created with different pulse energies can be discriminated clearly using ISO standard parameters, motif analysis and, over wide ranges of scales, using multiscale curvature analysis. Strong correlations can be found between the discharge pulse energies with which the surfaces were created and the texture characterization parameters calculated from the measurements. In six out of seven groups of ISO areal parameters, there are parameters that correlates well with discharge energy. Only spatial parameters could not be used to establish such functional relations, which may result from relatively small measurement area used to calculate autocorrelation function.

Most of modeling approaches, which explain the nature of electric discharge machining, focus on the creation of craters depending on the physical properties of material and controllable technological parameters [10,13,14,18,22]. The ability to characterize geometrical aspects of fabricated features becomes important step in the understanding of the interactions between surface and processing. Although most of ISO parameters correlate strongly with discharge energy, their ability to describe the aspects of craters morphology is generally weak or indirect. Height and functional groups include parameters that quantify, in various ways, height information focusing on *z*-component only. Spatial parameters involve calculation of auto-correlation function which reference to the craters’ geometry is vague. Hybrid parameters, which quantify both amplitude and spacing between heights, try to capture some geometric aspects of the surface (area and slope) but they consider a measured surface as a single entity not as a set of individual topographic features. Only feature parameters, related to dales or valleys, i.e., S5v, Sda and Sdv, have a direct association with crater depth, area and volume and, in addition, they correlate strongly with discharge energy. The similar statement can be formulated for the geometric parameters calculated using motif analysis, which is supported by the fact that they use same segmentation technique. Feature-based characterization is of particular importance in non-traditional manufacturing [47]. It provides additional perspective on the surface and supports better understanding of the phenomena governing manufacturing process and the interactions between controllable technological parameters and resulted surface topography [48]. This study proves that this is also a valuable approach for EDM. Geometrical properties of detected motifs, such as depth or volume, are easy to interpret in relation to their formation process, unlike average, root mean square roughness or skewness.

Multiscale curvature analysis, as presented in this study, is not specifically feature-based, but concentrates on the geometric characterization of shape when considering the surface as a composition of numerous overlapping craters. The key advantage of any multiscale analyses, unlike motifs and conventional studies, is that they can be useful in indicating the scales at which the correlations are the strongest. Knowledge of these scales could help to increase the understanding of the surface processing and function. It was noticed that absolute parameters, although they correlate strongly for the largest scales, do not vary significantly for the finest scales. Considering the three finest scales (<2.462 µm), coefficients of variation calculated for all analyzed surfaces do not exceed 10% for principal, mean and Gaussian curvature (average and SD). It might suggest that the created surfaces do not differ significantly at the finest scales, and also that the EDM process leads to the creation of fine-scale features similarly, regardless of the discharge energy. This might be supported by similar observations done by Hyde et al. [15] using areal multiscale analysis but different instrumentation, discharge energy level, material and outliers-removal method. What is more, curvature parameters start to correlate strongly with the discharge energy (*R*^2^ > 0.8) for scales starting from between 8 and 9 µm. This corresponds well to the average equivalent radius of detected motif (mean—1 × standard deviation ≈ 10 µm) for the sample created with lowest energy. Starting with those scales the curvature of craters is the most affected by the discharge energy and the fabricated microgeometry is the most adequately characterized as the sizes of features are best discerned at those scales. This follows the concept that scale could be enmeshed with size [3].

Strong correlations are observed for height parameters, which are most sensitive to longest wavelengths. This observation would appear to support similar, previously reported work/findings [15]. Spc (mean peak curvature) does not correlate well as it refers to the curvature at the finest scale as it is calculated for originally sampled data. Sdq and Sdr which might be related to multiscale areal method [5], when calculated also for the finest scale, correlate strongly with discharge energy only when regressed logarithmically. This might support our observation that the characterization of surface topography of EDM texture should be concentrated on the most accurate registration, filtration and analysis of large-scale features if relation between discharge energy is to be established.

Differences in physical processes that took essential role in the fabrication of surface finish might be analyzed through discrepancies in curvatures at similar scales on the surfaces manufactured by different discharge energies. Bartkowiak and Brown [7] found that for micro-EDM, there was a significant change in trend of topographic curvature versus discharge energies for surfaces manufactured with more than 1 µJ. Similar observations were made by Hyde et al. [15]. This study was done at significantly greater energies as well as different materials of both workpiece and electrode, and phenomenon of this kind was not observed. This might suggest that for the considered energies, the physical process that formed surface topography did not change its nature but rather the intensity. The aforementioned studies might have described the shift between micro and standard EDM process. In the former, electrostatic force might be a dominative factor in the creation of surface topography as there is simply not enough time for material to be moltened or vaporized and removed efficiently [28].

There is probably not a single parameter that can functionally describe the relations between technological parameters (discharge energy, current, voltage, gap, polarity) and fabricated surfaces, for all materials (electrode and workpiece), part shapes and machining conditions. Although some theoretical approaches are well known in conventional machining such as modeling Ra or Rz in turning and milling, the randomness and suddenness of electric discharges as well as complexity of physical phenomena make a development of analytical modeling for EDM rather challenging. Some undisclosed relations between VDI roughness and parameters of discharge are incorporated in the control systems of machine tools, as presented in this study. Therefore, establishing credible functional relations for most common materials and conditions are highly anticipated. Geometric characterization which focus on the morphology of craters is shown here to be a prospective candidate as it has a direct association with nature of the electric discharge machining.

Important aspect of surfaces is their designed functional behavior such as lubrication properties, gloss reflectance, corrosion resistance, load bearing capabilities, adhesion or wear. Basic profile or areal texture parameters correlate rarely with their performance parameters, or correlate only if narrow range of band-pass filter is applied [3,7]. Functional volume parameters certainly provide a better way of monitoring the effect of process parameters on the resulted surface texture. This can eventually help in defining proper machining conditions in order to fabricate surfaces according to functional needs [29]. Multiscale methods, including curvature, were found to be successful in establishing functional relations of those kinds for different processes such as friction, adhesion, fatigue, gloss and many more [3]. Motif analysis seem to be also prospective but further research should be conducted to fulfil its full potential. A primary focus should be given to establish functional relations between technological parameters of EDM and the functional behavior of fabricated surface through the adequate characterization of the topography.

From the metrological side, the main challenge for the measurement and dataset preparation steps was a reliable outliers removal process for FVM. As stated in Section 2.2, focus variation microscopy can produce surface- and method-specific outliers, that cannot be removed using standard removal procedures. The method used in this study was successful because of the small dimensions of the plateau-like formations, and therefore cannot be recommended as a general method for outliers removal. Considering all the aforementioned issues, the authors find using this method, in this particular study, justified. In addition, although there was a chance, that this method might affect roughness measurements. However, it introduced only insignificant changes, with its effect being similar to that of the λs -filter (microroughness filter).

Some other measurement techniques might be less susceptible to registering outliers on this kind of surface (locally smooth and reflective), such as CSI (coherent scanning interferometry) [36]. However, they are also burdened with both surface- and method-specific outliers, yet its hardware filtration is advanced, thus reducing the risk of unreliable measurements.

Another challenge of this study was to perform the correct filtration of the surface. A relatively small measurement area left little room for intuitive form and roughness evaluation. Therefore, a nesting index should be suited for the surface characteristic, originating from the manufacturing technology. The authors suggest that in this study, craters and their geometry belong to the roughness spectrum of the surface, thus the nesting index of 250 μm should be used. The resulting roughness surface contains most information regarding craters asperities. It must be noted that a larger measurement area would enable an easier choice of nesting index in future research. However, this poses another challenge for computational capabilities, since datasets derived from focus variation microscopes are quite large.

For the chosen nesting index areal parameters from the roughness surface are significantly influenced by the discharge energy. Waviness surface did not show any strong correlations. This might mean that the crucial information containing EDM-specific topographic features (craters) is in the roughness. Assuming a relatively large nesting index of 250 μm, it led to creation of residue surface of narrower bandwidth when compared to residual roughness. The maximum cut-off wavelength was limited by each measurement size of 323 μm × 323 μm. The primary surface, which contained all wavelengths was also rich in the information about craters geometry, what was evident in strong correlations with the discharge energy.

Conventional analyses using ISO parameters have the advantage that they are included in most commercial software and they can be evaluated with little knowledge of surface metrology principles (aside from noise and form removal). These make them used extensively by the industry and academia. Non-traditional characterization methods, such as motif and multiscale, are more complex and would require more expertise from the users. They will be appreciated once they add value by advancing the understanding of the relations between topographies and phenomena or if they can better exploit the acquired topographic information [2]. This could be facilitated by automatic, more intuitive and easy-to-use computer applications released for industrial and academic purposes. Motif analyses are included in commercial metrological software but they are limited to detection of dales and hills, whereas other geometric shapes can also be important signatures of manufacturing process.

## 5. Conclusions

The results show experimentally that the microgeometry of surfaces created by EDM is strongly affected by the discharge energy. This was proven by achieving strong correlations for geometric properties of fabricated features (height, area, diameter and volume) and their curvatures. In contrast to analysis of surface topography through ISO areal texture parameters, the presented approach extracted of information directly relating to the shape and size of topographic features of interest. This showed that geometric characterizations of process-specific surface formations were useful in determining functional relations with energy of electric discharges. Although, strong correlations with conventional parameters were also found, they miss the opportunity to study the effects of physical phenomena governing the creation of craters. Thus, geometric characterizations of crater morphology seem to be more natural and intuitive way of analysis of EDM surfaces. Further research in motifs and curvature versus technological parameters could promote better understanding and modeling of topographic response to its formation process.

Some detailed conclusions of this study can also be stated:

- Strong correlations (*R*^2^ > 0.9) were found between discharge energies and ISO parameters that were calculated for original surfaces (prior to S- or L-filtration but after morphological filtration) and S-surfaces (roughness). ISO standard parameters did not correlate well when computed for L-surfaces (waviness). This suggests that the creation of topographic features of larger dimensions is affected by the discharge energy. The dimension limit is constrained by cut-off wavelength of 250 µm. The characteristics of fine-scale surface features do not differ significantly. This is also supported by the outcome of multiscale curvature analysis which indicated that curvature correlated strongly also for larger scales.
- In the tested conditions, the surface is mostly affected and potentially controlled by discharge energy at larger scales which is associated with sizes of fabricated craters. For smaller scales, effect of machining with different parameters did not manifest itself.
- Strong correlations (*R*^2^ > 0.9) were also observed between motif parameters that characterized height, diameter, area and diameter of the detected motif, which might be associated with craters. This analysis, together with curvature and ISO areal parameters allow comprehensive characterization of surface microgeometry created by EDM.
- ISO areal parameters that describe peaks distributions exhibit higher coefficient of determination than others when regressed logarithmically. Correlations using a logarithmic model were also strong for curvature parameters.
- Registration of surface topography using focus variation microscopy leads to the occurrence of surface- and method-specific outliers which are hard to be removed using Gaussian filtration or thresholding. The application of a morphological filter proved to be successful in the outliers removal what was also evident to achieving strong correlations with discharge energy with parameters of three various types.

## Figures and Tables

**Table 1:** The percentage chemical composition of 1.2363 steel according to EN 10027-2.

C	Si	Mn	P	S	Cr	Mo	V
0.95–1.05	0.10–0.40	0.40–0.80	Max 0.030	Max 0.030	4.80–5.50	0.90–1.20	0.15–0.35

**Table 2:** Basic areal height parameters calculated for pre-electric discharge machined (EDM) (as-ground) surfaces according to ISO 25178 2. Data is presented as mean ± standard deviation for roughness and waviness.

Parameter	Sq (µm)	Sp (µm)	Sv (µm)	Sz (µm)	Sa (µm)
Roughness	0.541 ± 0.281	9.852 ± 4.866	3.752 ± 1.356	13.604 ± 5.448	0.418 ± 0.218
Waviness	0.232 ± 0.116	0.733 ± 0.389	0.577 ± 0.254	1.310 ± 0.635	0.184 ± 0.091

**Table 3:** Technological parameters of the finishing EDM.

Surface	U (V)	I (A)	T_on_ (µs)	T_off_ (µs)	Face Gap (mm)	Side Gap (mm)	Discharge Energy (µJ)	Theoretical VDI Class
S1	100	3.0	0.5	6.9	0.0126	0.126	150	16
S2	100	3.0	0.9	7.1	0.0150	0.0150	270	17
S3	100	3.0	1.8	7.5	0.0155	0.0155	540	18
S4	100	3.0	3.0	8.0	0.0160	0.0160	900	19
S5	100	3.0	4.8	8.8	0.0164	0.0164	1440	20
S6	100	3.0	7.3	9.9	0.0244	0.0206	2190	21
S7	100	3.0	10.9	11.5	0.0333	0.0253	3270	22
S8	100	3.0	16.1	13.8	0.0432	0.0304	4830	23
S9	100	3.2	22.8	16.6	0.0543	0.0361	7296	24
S10	100	3.6	26.3	19.7	0.0567	0.0376	9468	25

**Table 4:** Setup parameters of the focus variation microscope.

Parameter	Unit	Value
Magnification	-	50×
Area Dimensions	μm	323 × 323
Est. Vertical Resolution	μm	0.016
Est. Lateral Resolution	μm	2.31
Sampling intervals in x- and y-directions	μm	0.176

**Table 5:** ISO areal parameters examined in the study.

Parameter Group	Parameter Symbol
Height Parameters	Sq, Ssk, Sku, Sp, Sv, Sz, Sa
Functional Parameters	Smr, Smc, Sxp
Spatial Parameters	Sal, Str, Std
Hybrid Parameters	Sdq, Sdr
Functional Parameters (Volume)	Vm, Vv, Vmp, Vmc, Vvc, Vvv
Feature Parameters	Spd, Spc, S10z, S5p, S5v, Sda, Sha, Sdv, Shv
Functional Parameters (Stratified surfaces)	Sk, Spk, Svk, Smr1, Smr2

## Supplementary Materials

The following are available online at https://www.mdpi.com/1996-1944/13/17/3830/s1, Figure S1: renderings of representative regions of all ten analyzed surfaces, spreadsheet with ISO parameters calculated for primary, waviness and roughness surfaces.

## Appendix A

Appendix A contains supplementary information related to the sample preparations. This includes properties of graphite electrodes (Table A1) and EDM machine tool and dielectric fluid (Table A2).

**Table A1:** Graphite electrode parameters.

Properties	Units	Test Standard	Values
Average grain size	µm	ISO 13320	3
Bulk density	g/cm^3^	DIN IEC 60413/204	1.88
Open porosity	Vol. %	DIN 66133	10
Medium pore entrance diameter	µm	DIN 66133	0.6
Ambient temperature	cm^2^/s	DIN 51935	0.01
Hardness	HR _5_/_100_	DIN IEC 60413/303	105
Resistivity	µΩm	DIN IEC 60413/402	13
Flexural strength	MPa	DIN IEC 60413/501	85
Compressive strength	MPa	DIN 51910	170
Dynamic modulus of elasticity	MPa	DIN 51915	13.5 × 10^3^
Thermal expansion (20–200 °C)	K^−1^	DIN 51909	4.7 × 10^−6^
Thermal conductivity (20 °C)	Wm^−1^K^−1^	DIN 51908	105
Ash content	ppm	DIN 51903	200

**Table A2:** Machine o dielectric fluid parameters.

Properties	Units	Values
Machine		
Architecture		C-frame/Fixed table/Drop tank
X, Y, Z travel	mm	400 × 300 × 350
X, Y axes speed	m/min	6
Z axis speed	m/min	15
Positioning resolution	µm	0.1
Work tank size	Mm	900 × 630 × 350
Work table size	Mm	600 × 400
Max. machining current	A	80
Best surface finish Ra	µm	0.08
**Dielectric fluid**		
Color		Straw yellow
Kinematic viscosity (20 °C/40 °C)	mm^2^/s	5.0/3.0
Density	kg/l	0.77

## Appendix B

Appendix B presents supplementary information related to the regression analyses for ISO 25178 areal parameters (Table A3) and motif parameters (Table A4). Both of parameter groups were regressed using linear and logarithmic regression. Coefficients of determination (*R*^2^) is given for all analyzed parameters.

**Table A3:** Coefficients of determination (*R*^2^) between discharge energies and ISO 25178 areal parameters calculated using linear and logarithmic regressions for primary surfaces. Please see the standard for additional settings given in the table.

Parameter	*R*^2^ for Linear Regression	*R*^2^ for Logarithmic Regression
Sq	0.930	0.905
Ssk	0.643	0.291
Sku	0.016	0.001
Sp	0.760	0.961
Sv	0.918	0.849
Sz	0.891	0.934
Sa	0.930	0.899
Smr (c = 1 µm under highest peak)	0.024	0.207
Smc (p = 10%)	0.911	0.923
Sxp (p = 50%, q = 97.5%)	0.953	0.846
Sal (s = 0.2)	0.014	0.117
Str (s = 0.2)	0.566	0.531
Std (reference = 0°)	0.113	0.115
Sdq	0.796	0.929
Sdr	0.825	0.916
Vm (p = 10%)	0.790	0.973
Vv (p = 10%)	0.909	0.927
Vmp (p = 10%)	0.790	0.973
Vmc (p = 10%, q = 80%)	0.941	0.881
Vvc (p = 10%, q = 80%)	0.899	0.933
Vvv (p = 80%)	0.952	0.838
Spd (trimming = 0.75%)	0.661	0.947
Spc (trimming = 0.75%)	0.302	0.335
S10z (trimming = 0.75%)	0.853	0.942
S5p (trimming = 0.75%)	0.557	0.901
S5v (trimming = 0.75%)	0.932	0.838
Sda (trimming = 0.75%)	0.932	0.904
Sha (trimming = 0.75%)	0.938	0.890
Sdv (trimming = 0.75%)	0.963	0.716
Shv (trimming = 0.75%)	0.959	0.836
Sk (unfiltered)	0.923	0.887
Spk (unfiltered)	0.772	0.980
Svk unfiltered)	0.953	0.832
Smr1 (unfiltered)	0.292	0.032
Smr2 (unfiltered)	0.631	0.398

**Table A4:** Coefficients of determination (*R*^2^) between discharge energies and motif parameters calculated using linear and logarithmic regressions.

Motif Parameter	Statistics	*R*^2^ for Linear Regression	*R*^2^ for Logarithmic Regression
Number of motifs	-	0.874	0.936
Height	Mean	0.979	0.766
Height	SD	0.976	0.784
Area	Mean	0.986	0.826
Area	SD	0.975	0.841
Volume	Mean	0.965	0.660
Volume	SD	0.961	0.665
Mean diameter	Mean	0.971	0.861
Mean diameter	SD	0.955	0.877
Minimal diameter	Mean	0.963	0.867
Minimal diameter	SD	0.953	0.846
Maximal diameter	Mean	0.964	0.871
Maximal diameter	SD	0.965	0.891
