# Supplementary material for: Analysis of Surface Microgeometry Created by Electric Discharge Machining
Source: Materials (Basel). 2020 Aug 30;13(17):3830. doi: 10.3390/ma13173830 (PMC7504530; doi:10.3390/ma13173830)
Supplement: Supplementary file 1 [file materials-13-03830-s001.pdf]

materials

Supplementary Information

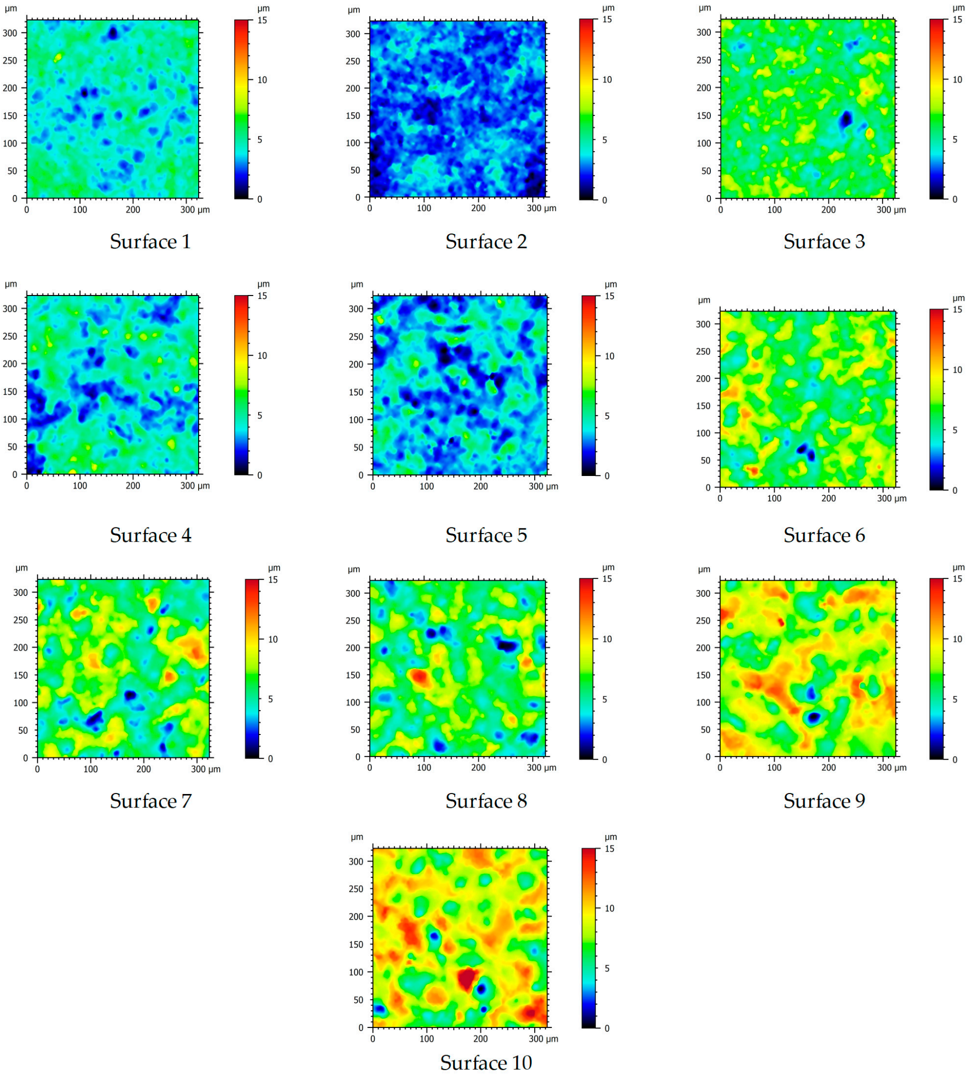

Figure S1. Renderings of representative regions of all ten analyzed surfaces.

© 2020 by the authors. Submitted for possible open access publication under the terms and conditions of the Creative Commons Attribution (CC BY) license (http://creativecommons.org/licenses/by/4.0/).
